# Improvement in sleep latency with extended-release once-nightly sodium oxybate for the treatment of adults with narcolepsy: Analysis from the phase 3 REST-ON clinical trial

**DOI:** 10.1016/j.sleepx.2024.100113

**Published:** 2024-05-03

**Authors:** Michael J. Thorpy, Clete A. Kushida, Richard Bogan, John Winkelman, Maurice M. Ohayon, Colin M. Shapiro, Jennifer Gudeman

**Affiliations:** a Albert Einstein College of Medicine, New York, NY, USA; b Stanford University School of Medicine, Stanford, CA, USA; c Medical University of South Carolina, Charleston, SC, USA; d Massachusetts General Hospital, Boston, MA, USA; e Stanford University School of Medicine, Palo Alto, CA, USA; f University of Toronto, Toronto, ON, Canada; g Avadel Pharmaceuticals, Chesterfield, MO, USA

**Keywords:** Narcolepsy, Excessive daytime sleepiness, Sodium oxybate, Extended release, Once nightly, FT218

## Abstract

**Background:**

In the REST-ON clinical trial (NCT02720744), mean sleep latency on the Maintenance of Wakefulness Test (MWT) was significantly improved with extended-release once-nightly sodium oxybate (ON-SXB) vs placebo (*P* < 0.001) in participants with narcolepsy. This post hoc analysis assessed response to treatment and improvement in excessive daytime sleepiness.

**Methods:**

Participants with narcolepsy aged ≥16 years were randomized 1:1 to receive ON-SXB (4.5 g, week 1; 6 g, weeks 2–3; 7.5 g, weeks 3–8; and 9 g, weeks 9–13) or placebo. Mean sleep latency on the MWT was measured across 5 trials of ≤30 min each. Post hoc assessments included percentage of participants whose sleep latency improved ≥5, ≥10, ≥15, and ≥20 min and with a mean sleep latency of 30 min.

**Results:**

Significantly more participants receiving ON-SXB vs placebo experienced increased mean sleep latency ≥5 min (all doses *P* < 0.001), ≥10 min (all doses *P* < 0.001), ≥15 min (6 and 7.5 g, *P* < 0.001; 9 g, *P* < 0.01), and ≥20 min (6 g, *P* < 0.01; 7.5 g, *P* < 0.001; 9 g, *P* < 0.05). More participants receiving ON-SXB had mean sleep latency of 30 min vs placebo (6 g, 5.7 % vs 0 %, respectively [*P* < 0.05]; 7.5 g, 10.5 % vs 1.3 % [*P* < 0.05]; 9 g, 13.2 % vs 5.1 % [*P* = 0.143]).

**Conclusions:**

Significantly more participants who received ON-SXB experienced increased mean sleep latency ≥5 to ≥20 min; at the 2 highest doses, >10 % remained awake for the entirety of the MWT. ON-SXB offers a once-at-bedtime treatment option for adults with narcolepsy.

## Highlights

• Once-nightly sodium oxybate (ON-SXB) is an extended-release oxybate formulation.
• Clinically meaningful increases in sleep latency occurred with all ON-SXB doses.
• Some participants were able to remain awake for the entirety of the 30-min test.
• Daytime sleepiness was significantly improved in patients with narcolepsy.
• ON-SXB offers a once-at-bedtime option for patients with narcolepsy.

## 1 Introduction

Narcolepsy is a rare, chronic neurologic sleep disorder that is characterized by excessive daytime sleepiness (EDS); hypnagogic and/or hypnopompic hallucinations; fragmented sleep; sleep paralysis; and, in some individuals, cataplexy (narcolepsy type 1 [NT1]) [1,2]. EDS, the hallmark symptom that all people with narcolepsy experience, is particularly disabling and imposes a substantial burden [3].

Sodium oxybate (SXB), the sodium salt of γ-hydroxybutyrate, has demonstrated efficacy across multiple narcolepsy symptom domains, including EDS and cataplexy [2,[4], [5], [6]]. Based on the findings of several randomized controlled studies, the American Academy of Sleep Medicine (AASM) has strongly recommended SXB as a treatment for EDS and cataplexy in adults with narcolepsy [6]. Previously, all approved oxybate formulations were immediate release and required twice-nightly dosing, which requires individuals with narcolepsy to awaken 2.5–4 h after the first dose to take a second dose to cover a full night of sleep [7,8]. Once-nightly sodium oxybate (ON-SXB, FT218; LUMRYZ™ [sodium oxybate] for extended-release oral suspension, Avadel Pharmaceuticals) is an extended-release formulation of SXB that was approved by the US Food and Drug Administration for the treatment of cataplexy or EDS in adults with narcolepsy in May 2023 [9,10].

Safety and efficacy of ON-SXB were evaluated in the phase 3, randomized, placebo-controlled REST-ON clinical trial (NCT02720744) [11]. In the REST-ON trial, ON-SXB demonstrated statistically significant improvements vs placebo (*P* < 0.001) for all 3 coprimary endpoints, which included mean sleep latency on the Maintenance of Wakefulness Test (MWT), the Clinical Global Impression of Improvement rating of much/very much improved, and the number of weekly cataplexy attacks, for all evaluated doses (6, 7.5, and 9 g) [11]. In this post hoc analysis of the REST-ON clinical trial, response to treatment and improvement in EDS were further characterized.

## 2 Methods

### 2.1 Study design and participants

Detailed methods of the phase 3 REST-ON clinical trial (NCT02720744), including study design, inclusion/exclusion criteria, primary outcomes, and statistical analyses have been previously reported [11]. Briefly, individuals aged ≥16 years diagnosed with NT1 or narcolepsy type 2 (NT2), mean sleep latency on MWT <11 min following baseline polysomnography, Epworth Sleepiness Scale (ESS) score >10, and for NT1 a mean of ≥8 cataplexy attacks per week during the screening period were eligible for inclusion. Participants were stratified by narcolepsy type (NT1 or NT2) and randomized 1:1 to receive ON-SXB or placebo according to the following schedule: 4.5 g for 1 week, 6 g for 2 weeks, 7.5 g for 5 weeks, and 9 g for 5 weeks (13 weeks of treatment). Participants could continue to receive concomitant stimulant therapy or other wake-promoting agents throughout the study if they had been on a stable regimen for ≥3 weeks before study entry, and they maintained the same stimulant regimen throughout the entire study period.

The study protocol was approved by the sites’ institutional review boards, and each participant (and legal representative for those aged <18 y) provided written informed consent before trial participation. The study was conducted according to the ethical principles of the Good Clinical Practice guidelines, the International Council for Harmonisation guidelines, and the Declaration of Helsinki, as well as any applicable national and local laws and regulatory requirements [11].

### 2.2 Sleep latency

Mean sleep latency was measured with the MWT across 5 trials of up to 30 min each. The MWT was performed at baseline and at weeks 3 (6-g dose), 8 (7.5-g dose), and 13 (9-g dose) of treatment. Post hoc assessments included the percentage of participants in each treatment arm whose sleep latency improved ≥5, ≥10, ≥15, and ≥20 min from baseline on the MWT and the percentage of participants who had a mean sleep latency of 30 min.

### 2.3 Statistical analysis

Efficacy was assessed in the modified intent-to-treat population, which comprised all randomized participants with ≥1 efficacy measurement after receiving the 6-g dose (ON-SXB or placebo). Fisher exact test was used to calculate 2-sided *P* values for the percentage of participants with ≥5-, ≥10-, ≥15-, and ≥20-min improvement from baseline on the MWT and the percentage of participants who had a mean sleep latency of 30 min on the MWT across five 30-min trials averaged over the test day.

## 3 Results

### 3.1 Participants

Of the 222 participants randomized to receive ON-SXB or placebo in REST-ON, 212 (ON-SXB, n = 107; placebo, n = 105) received ≥1 dose of study drug, and 148 (70 %) completed the study (ON-SXB, n = 69 [65 %]; placebo, n = 79 [75 %]). The mean (SD) participant age was 31.2 (11.0) years, 144 (68 %) were women, 160 (75 %) were white, 162 (76 %) had NT1, and 50 (24 %) had NT2. Adherence rates were 83 % and 91 % for participants who received ON-SXB and placebo, respectively.

### 3.2 Sleep latency

**Fig. 1:**
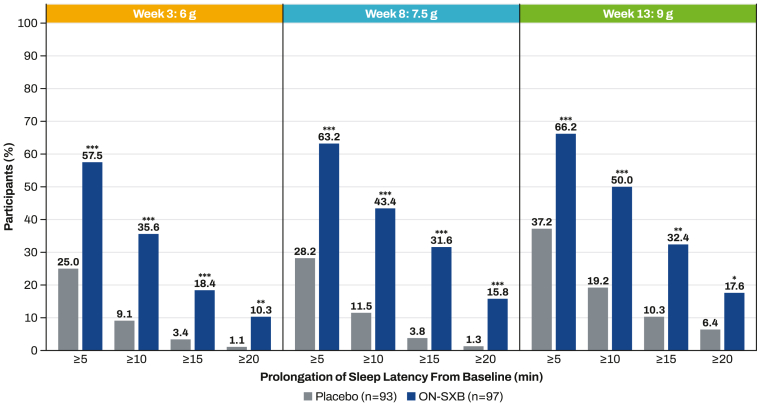
Improvement in mean sleep latency on the Maintenance of Wakefulness Test (mITT population). mITT, modified intent to treat; MWT, Maintenance of Wakefulness Test; ON-SXB, once-nightly sodium oxybate. *P < 0.05; **P < 0.01; ***P < 0.001 vs placebo.

At baseline, the mean (SD) sleep latency on the MWT was 5.0 (3.2) minutes in the ON-SXB arm and 4.7 (2.6) minutes in the placebo arm. Compared to placebo, a significantly greater proportion of participants in the ON-SXB treatment arm had increased mean sleep latency from baseline of ≥5 min (all doses, *P* < 0.001), ≥10 min (all doses, *P* < 0.001), ≥15 min (6 and 7.5 g, *P* < 0.001; 9 g, *P* < 0.01), and ≥20 min (6 g, *P* < 0.01; 7.5 g, *P* < 0.001; 9 g, *P* < 0.05) (Fig. 1). Significantly more participants receiving ON-SXB 6 and 7.5 g vs placebo remained awake for the entire 30 min of the MWT (6 g, 5.7 % vs 0 %, respectively; 7.5 g, 10.5 % vs 1.3 %; both *P* < 0.05) (Table 1). A higher percentage of participants had a mean MWT of 30 min for the 9-g dose of ON-SXB (13.2 %) compared to placebo (5.1 %; *P* = 0.143).

**Table 1:** Responder analysis: Maintenance of Wakefulness Test (modified intent-to-treat population).

	Week 3		Week 8		Week 13	
Mean Sleep Latency	Placebo (n = 88)	ON-SXB 6 g (n = 87)	Placebo (n = 78)	ON-SXB 7.5 g (n = 76)	Placebo (n = 78)	ON-SXB 9 g (n = 68)
30 min, n (%)	0	5 (5.7)	1 (1.3)	8 (10.5)	4 (5.1)	9 (13.2)
*P* value	<0.05		<0.05		0.143	

ON-SXB, once-nightly sodium oxybate. n is the number of participants in the trial at the end of the treatment period.

## 4 Discussion

The phase 3 REST-ON clinical trial showed that treatment with ON-SXB significantly improved mean sleep latency on the MWT vs placebo at all prespecified evaluated doses, which was a coprimary endpoint (*P* < 0.001) [11]. In these post hoc analyses, significantly more participants who received ON-SXB experienced increased sleep latency on the MWT ranging from ≥5 to ≥20 min compared to placebo. Improvement was evident as early as week 3 at the 6-g dose and increased with the 7.5-g dose at week 8 and the 9-g dose at week 13. In addition, more participants receiving ON-SXB remained awake for the entire 30-min MWT with all ON-SXB doses; results were significant vs placebo at week 3 (6-g dose) and week 8 (7.5-g dose).

Excessive daytime sleepiness imposes a substantial burden on individuals with narcolepsy [3]. In addition to MWT, improvement in EDS was also assessed in REST-ON using the patient-reported ESS (secondary endpoint) [11]. Participants receiving ON-SXB had significantly greater improvement from baseline vs placebo in ESS score at all doses (6, 7.5, and 9 g; all *P* < 0.001) [11]. Clinical significance thresholds for improvement in EDS were classified by AASM in their recent clinical practice guidelines as an increase from baseline vs placebo of ≥2 min on the MWT and a ≥2-point decrease in ESS score [12]. These thresholds for improvement in EDS were met after treatment with ON-SXB on the MWT and the ESS [13].

There are limitations to consider, particularly given that these analyses were post hoc. An increasing placebo response was observed during the trial and may have occurred because of an expected dose response with the required dose escalation at defined points in the trial. This placebo effect may contribute to the lack of statistical significance between the ON-SXB and placebo treatment groups at the 30-min MWT threshold, as 4 participants in the placebo group stayed awake across all 5 tests compared to 9 participants in the ON-SXB group at week 13. Additionally, it is possible that the 6-g dose would have increased the mean sleep latency on the MWT further if dosing had continued past week 3; even with the lowest dose and earliest time assessment, the 6-g dose demonstrated significant efficacy for both the prespecified endpoint and these post hoc analyses compared to placebo.

## 5 Conclusions

ON-SXB is an efficacious treatment for EDS in individuals with narcolepsy. These findings suggest that the magnitude of response with ON-SXB was clinically meaningful and may facilitate patient counseling and help set treatment expectations with ON-SXB. ON-SXB offers a once-at-bedtime treatment option for adults with narcolepsy.

## Funding

This study was funded by 10.13039/100005646Avadel Pharmaceuticals (Chesterfield, MO), which was involved in the study design; in the collection, analysis and interpretation of data; in the writing of the report; and in the decision to submit the article for publication.

## CRediT authorship contribution statement

**Michael J. Thorpy:** Writing – review & editing, Formal analysis. **Clete A. Kushida:** Writing – review & editing, Formal analysis. **Richard Bogan:** Writing – review & editing, Formal analysis. **John Winkelman:** Writing – review & editing, Formal analysis. **Maurice M. Ohayon:** Writing – review & editing, Formal analysis. **Colin M. Shapiro:** Writing – review & editing, Formal analysis. **Jennifer Gudeman:** Writing – review & editing, Formal analysis.

## Declaration of competing interest

The authors declare the following financial interests/personal relationships which may be considered as potential competing interests:

Michael J. Thorpy reports a relationship with Axsome Therapeutics that includes: consulting or advisory. Michael J. Thorpy reports a relationship with Balance Therapeutics that includes: consulting or advisory. Michael J. Thorpy reports a relationship with Eisai that includes: consulting or advisory. Michael J. Thorpy reports a relationship with Avadel Pharmaceuticals that includes: consulting or advisory. Michael J. Thorpy reports a relationship with Harmony BioSciences that includes: consulting or advisory. Michael J. Thorpy reports a relationship with 10.13039/100011096Jazz Pharmaceuticals that includes: consulting or advisory. Michael J. Thorpy reports a relationship with NLS Pharmaceuticals that includes: consulting or advisory. Michael J. Thorpy reports a relationship with Suven Life Sciences Ltd. that includes: consulting or advisory. Michael J. Thorpy reports a relationship with 10.13039/100008373Takeda Pharmaceutical Co that includes: consulting or advisory. Clete A. Kushida reports a relationship with Avadel Pharmaceutical that includes: consulting. Clete A. Kushida reports a relationship with XW Pharma that includes: consulting. Richard Bogan reports a relationship with WaterMark Medical and Health Humming LLC that includes: equity or stocks. Richard Bogan reports a relationship with WaterMark Medical that includes: board membership. Richard Bogan reports a relationship with 10.13039/100011096Jazz Pharmaceuticals that includes: consulting or advisory, funding grants, and speaking and lecture fees. Richard Bogan reports a relationship with Harmony BioSciences that includes: consulting or advisory and speaking and lecture fees. Richard Bogan reports a relationship with 10.13039/100008373Takeda Pharmaceutical Co. that includes: consulting or advisory and funding grants. Richard Bogan reports a relationship with Avadel Pharmaceuticals that includes: consulting or advisory and funding grants. Richard Bogan reports a relationship with Oventus that includes: consulting or advisory. Richard Bogan reports a relationship with BresoTec that includes: funding grants. Richard Bogan reports a relationship with 10.13039/100004326Bayer that includes: funding grants. Richard Bogan reports a relationship with 10.13039/501100016198Idorsia that includes: funding grants. Richard Bogan reports a relationship with Suven Life Sciences Ltd. that includes: funding grants. Richard Bogan reports a relationship with Balance Therapeutics that includes: funding grants. Richard Bogan reports a relationship with 10.13039/100010902Vanda that includes: funding grants. Richard Bogan reports a relationship with 10.13039/100004334Merck that includes: funding grants. Richard Bogan reports a relationship with 10.13039/501100003769Eisai that includes: funding grants and speaking and lecture fees. Richard Bogan reports a relationship with Fresca that includes: funding grants. Richard Bogan reports a relationship with 10.13039/100013410LivaNova that includes: funding grants. Richard Bogan reports a relationship with 10.13039/100004337Roche that includes: funding grants. Richard Bogan reports a relationship with Sommetrics that includes: funding grants. John Winkelman reports a relationship with Avadel Pharmaceuticals that includes: consulting or advisory. John Winkelman reports a relationship with Emalex Biosciences that includes: consulting or advisory. John Winkelman reports a relationship with Noctrix Health that includes: consulting or advisory. John Winkelman reports a relationship with Disc Medicine that includes: consulting or advisory. John Winkelman reports a relationship with 10.13039/501100016198Idorsia Pharmaceuticals that includes: consulting or advisory. John Winkelman reports a relationship with 10.13039/100004334Merck & Co. that includes: funding grants. John Winkelman reports a relationship with 10.13039/100016473American Regent that includes: funding grants. John Winkelman reports a relationship with 10.13039/100000026NIDA that includes: funding grants. John Winkelman reports a relationship with 10.13039/100004087RLS Foundation that includes: funding grants. John Winkelman reports a relationship with Baszucki Brain Research Fund that includes: funding grants. Maurice M. Ohayon reports a relationship with Avadel Pharmaceuticals that includes: consulting or advisory. Maurice M. Ohayon reports a relationship with 10.13039/100008373Takeda Pharmaceutical Co. that includes: consulting or advisory. Maurice M. Ohayon reports a relationship with Jazz Pharmaceuticals that includes: consulting or advisory. Colin M. Shapiro reports a relationship with Avadel Pharmaceuticals that includes: consulting or advisory and speaking and lecture fees. Colin M. Shapiro reports a relationship with 10.13039/100011096Jazz Pharmaceuticals that includes: consulting or advisory and speaking and lecture fees. Jennifer Gudeman reports a relationship with Avadel Pharmaceuticals that includes: employment. If there are other authors, they declare that they have no known competing financial interests or personal relationships that could have appeared to influence the work reported in this paper.
